# Clinical significance of the estimation of pulmonary-right ventricular uncoupling in patients with transthyretin amyloid cardiomyopathy

**DOI:** 10.1093/ehjimp/qyae113

**Published:** 2025-01-17

**Authors:** Hiroki Usuku, Eiichiro Yamamoto, Kasumi Miyazaki, Ryudai Higashi, Atsushi Nozuhara, Fumi Oike, Naoto Kuyama, Noriaki Tabata, Masanobu Ishii, Shinsuke Hanatani, Tadashi Hoshiyama, Hisanori Kanazawa, Daisuke Sueta, Yuichiro Arima, Seitaro Oda, Hiroaki Kawano, Yasushi Matsuzawa, Yasuhiro Izumiya, Mitsuharu Ueda, Yasuhito Tanaka, Kenichi Tsujita

**Affiliations:** Department of Laboratory Medicine, Kumamoto University Hospital, 1-1-1 Honjo, Chuo-ku, Kumamoto 860-8556, Japan; Department of Cardiovascular Medicine, Graduate School of Medical Sciences, Kumamoto University, 1-1-1 Honjo, Chuo-ku, Kumamoto 860-8556, Japan; Center of Metabolic Regulation of Healthy Aging, Faculty of Life Sciences, Kumamoto University, 1-1-1 Honjo, Chuo-ku, Kumamoto 860-8556, Japan; Department of Cardiovascular Medicine, Graduate School of Medical Sciences, Kumamoto University, 1-1-1 Honjo, Chuo-ku, Kumamoto 860-8556, Japan; Center of Metabolic Regulation of Healthy Aging, Faculty of Life Sciences, Kumamoto University, 1-1-1 Honjo, Chuo-ku, Kumamoto 860-8556, Japan; Department of Laboratory Medicine, Kumamoto University Hospital, 1-1-1 Honjo, Chuo-ku, Kumamoto 860-8556, Japan; Department of Cardiovascular Medicine, Graduate School of Medical Sciences, Kumamoto University, 1-1-1 Honjo, Chuo-ku, Kumamoto 860-8556, Japan; Center of Metabolic Regulation of Healthy Aging, Faculty of Life Sciences, Kumamoto University, 1-1-1 Honjo, Chuo-ku, Kumamoto 860-8556, Japan; Department of Cardiovascular Medicine, Graduate School of Medical Sciences, Kumamoto University, 1-1-1 Honjo, Chuo-ku, Kumamoto 860-8556, Japan; Center of Metabolic Regulation of Healthy Aging, Faculty of Life Sciences, Kumamoto University, 1-1-1 Honjo, Chuo-ku, Kumamoto 860-8556, Japan; Department of Cardiovascular Medicine, Graduate School of Medical Sciences, Kumamoto University, 1-1-1 Honjo, Chuo-ku, Kumamoto 860-8556, Japan; Center of Metabolic Regulation of Healthy Aging, Faculty of Life Sciences, Kumamoto University, 1-1-1 Honjo, Chuo-ku, Kumamoto 860-8556, Japan; Department of Cardiovascular Medicine, Graduate School of Medical Sciences, Kumamoto University, 1-1-1 Honjo, Chuo-ku, Kumamoto 860-8556, Japan; Center of Metabolic Regulation of Healthy Aging, Faculty of Life Sciences, Kumamoto University, 1-1-1 Honjo, Chuo-ku, Kumamoto 860-8556, Japan; Department of Cardiovascular Medicine, Graduate School of Medical Sciences, Kumamoto University, 1-1-1 Honjo, Chuo-ku, Kumamoto 860-8556, Japan; Center of Metabolic Regulation of Healthy Aging, Faculty of Life Sciences, Kumamoto University, 1-1-1 Honjo, Chuo-ku, Kumamoto 860-8556, Japan; Department of Cardiovascular Medicine, Graduate School of Medical Sciences, Kumamoto University, 1-1-1 Honjo, Chuo-ku, Kumamoto 860-8556, Japan; Center of Metabolic Regulation of Healthy Aging, Faculty of Life Sciences, Kumamoto University, 1-1-1 Honjo, Chuo-ku, Kumamoto 860-8556, Japan; Department of Cardiovascular Medicine, Graduate School of Medical Sciences, Kumamoto University, 1-1-1 Honjo, Chuo-ku, Kumamoto 860-8556, Japan; Center of Metabolic Regulation of Healthy Aging, Faculty of Life Sciences, Kumamoto University, 1-1-1 Honjo, Chuo-ku, Kumamoto 860-8556, Japan; Department of Cardiovascular Medicine, Graduate School of Medical Sciences, Kumamoto University, 1-1-1 Honjo, Chuo-ku, Kumamoto 860-8556, Japan; Center of Metabolic Regulation of Healthy Aging, Faculty of Life Sciences, Kumamoto University, 1-1-1 Honjo, Chuo-ku, Kumamoto 860-8556, Japan; Department of Cardiovascular Medicine, Graduate School of Medical Sciences, Kumamoto University, 1-1-1 Honjo, Chuo-ku, Kumamoto 860-8556, Japan; Center of Metabolic Regulation of Healthy Aging, Faculty of Life Sciences, Kumamoto University, 1-1-1 Honjo, Chuo-ku, Kumamoto 860-8556, Japan; Department of Cardiovascular Medicine, Graduate School of Medical Sciences, Kumamoto University, 1-1-1 Honjo, Chuo-ku, Kumamoto 860-8556, Japan; Center of Metabolic Regulation of Healthy Aging, Faculty of Life Sciences, Kumamoto University, 1-1-1 Honjo, Chuo-ku, Kumamoto 860-8556, Japan; Department of Cardiovascular Medicine, Graduate School of Medical Sciences, Kumamoto University, 1-1-1 Honjo, Chuo-ku, Kumamoto 860-8556, Japan; Center of Metabolic Regulation of Healthy Aging, Faculty of Life Sciences, Kumamoto University, 1-1-1 Honjo, Chuo-ku, Kumamoto 860-8556, Japan; Department of Diagnostic Radiology, Faculty of Life Sciences, Kumamoto University, 1-1-1 Honjo, Chuo-ku, Kumamoto 860-8556, Japan; Department of Cardiovascular Medicine, Graduate School of Medical Sciences, Kumamoto University, 1-1-1 Honjo, Chuo-ku, Kumamoto 860-8556, Japan; Center of Metabolic Regulation of Healthy Aging, Faculty of Life Sciences, Kumamoto University, 1-1-1 Honjo, Chuo-ku, Kumamoto 860-8556, Japan; Department of Cardiovascular Medicine, Graduate School of Medical Sciences, Kumamoto University, 1-1-1 Honjo, Chuo-ku, Kumamoto 860-8556, Japan; Center of Metabolic Regulation of Healthy Aging, Faculty of Life Sciences, Kumamoto University, 1-1-1 Honjo, Chuo-ku, Kumamoto 860-8556, Japan; Department of Cardiovascular Medicine, Graduate School of Medical Sciences, Kumamoto University, 1-1-1 Honjo, Chuo-ku, Kumamoto 860-8556, Japan; Center of Metabolic Regulation of Healthy Aging, Faculty of Life Sciences, Kumamoto University, 1-1-1 Honjo, Chuo-ku, Kumamoto 860-8556, Japan; Center of Metabolic Regulation of Healthy Aging, Faculty of Life Sciences, Kumamoto University, 1-1-1 Honjo, Chuo-ku, Kumamoto 860-8556, Japan; Department of Neurology, Graduate School of Medical Sciences, Kumamoto University, 1-1-1 Honjo, Chuo-ku, Kumamoto 860-8556, Japan; Department of Laboratory Medicine, Kumamoto University Hospital, 1-1-1 Honjo, Chuo-ku, Kumamoto 860-8556, Japan; Department of Cardiovascular Medicine, Graduate School of Medical Sciences, Kumamoto University, 1-1-1 Honjo, Chuo-ku, Kumamoto 860-8556, Japan; Center of Metabolic Regulation of Healthy Aging, Faculty of Life Sciences, Kumamoto University, 1-1-1 Honjo, Chuo-ku, Kumamoto 860-8556, Japan

**Keywords:** transthyretin amyloid cardiomyopathy, pulmonary-right ventricular uncoupling, two-dimensional speckle tracking imaging

## Abstract

**Aims:**

There are few data on the prognostic impact of pulmonary-right ventricular (RV) uncoupling in patients with wild-type transthyretin amyloid cardiomyopathy (ATTRwt-CM).

**Methods and results:**

Among the 174 patients who were diagnosed with ATTRwt-CM at Kumamoto University Hospital from 2002 to 2021, 143 patients who met the current Japanese guideline and had sufficient information for two-dimensional speckle tracking echocardiography were retrospectively analysed. During a median follow-up of 1209 days, 39 cardiac deaths occurred. Compared with patients in the non-event group, those in the cardiac death group were significantly older (79.3 ± 6.7 vs. 76.4 ± 6.2, respectively; *P* < 0.05). Additionally, RV global longitudinal strain (RV-GLS)/systolic pulmonary artery pressure (sPAP), an index of pulmonary-RV uncoupling, was significantly lower in patients in the cardiac death group vs. the non-event group [0.29 (0.18–0.35) vs. 0.40 (0.29–0.57), *P* < 0.01]. Multivariate Cox proportional hazards regression analysis demonstrated that RV-GLS/sPAP was significantly associated with cardiac death after adjusting for tricuspid annular plane systolic excursion/sPAP (*P* < 0.01), sPAP (*P* < 0.05), and conventional prognostic factors including age and hospitalization for heart failure (<0.01), laboratory finding including high-sensitivity cardiac troponin T, and B-type natriuretic peptide (*P* < 0.01). Receiver operating characteristic analysis showed that the area under the curve for RV-GLS/sPAP for cardiac death was 0.72 and that the best cut off value for RV-GLS/sPAP was 0.34 (sensitivity, 76%; specificity, 65%). In the Kaplan–Meier analysis, patients with ATTRwt-CM who had low vs. high RV-GLS/sPAP (cut-off value 0.34) had a significantly higher probability of cardiac death (*P* < 0.01).

**Conclusion:**

Pulmonary-RV uncoupling has significantly higher prognostic value compared with conventional prognostic factors in ATTRwt-CM.

## Graphical Abstract

**Graphical Abstract:**
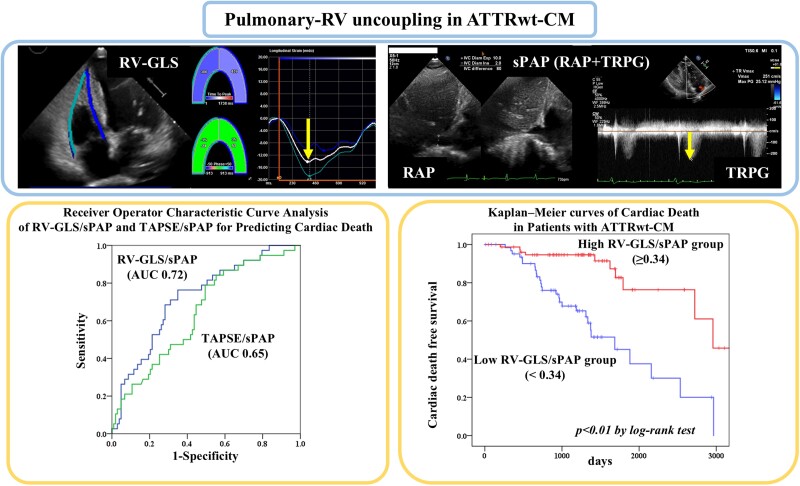

## Introduction

Transthyretin (TTR) amyloid cardiomyopathy (ATTR-CM) is becoming increasingly recognized because of population aging, advancements in the understanding of the disease pathobiology, and the potential benefits of emerging therapies.^1,2^ ATTR-CM is further classified into two subtypes by the presence or absence of *TTR* gene mutations: mutant ATTR-CM and wild-type ATTR-CM (ATTRwt-CM). Because ATTRwt-CM leads to repeat hospitalizations and cardiac death,^3^ identification of vulnerable patients with ATTRwt-CM at high risk of cardiac death is important, clinically. Several studies have shown that high-sensitivity cardiac troponin T (hs-cTnT) and B-type natriuretic peptide (BNP) are useful prognostic markers for patients with ATTRwt-CM.^4,5^ However, conventional echocardiographic parameters, such as left ventricular (LV) wall thickness, LV mass, and diastolic function, were not independent predictors of survival in these studies.

Two-dimensional strain analysis with speckle tracking echocardiography has recently been used to detect myocardial deformation.^6^ Left atrial (LA) strain was known to be useful to differentiate amyloid cardiomyopathy and hypertrophic cardiomyopathy.^7^ We previously focused on right ventricular (RV) function and reported that RV global longitudinal strain (RV-GLS) was a significant prognostic factor in patients with ATTRwt-CM.^8^ Additionally, several new factors, such as pulmonary-right ventricular (RV) uncoupling, LA stiffness, and the visually assessed time difference between mitral valve (MV) and tricuspid valve (TV) opening (VMT score), are echocardiographic factors that predict the prognosis in patients with various cardiovascular diseases.^9–11^ However, in patients with ATTRwt-CM, the prognostic utilities of these factors were not fully evaluated.

## Methods

### Study population

In total, 174 patients were diagnosed with ATTRwt-CM at Kumamoto University Hospital from December 2002 to December 2021. Of these patients, 24 were excluded from this study because their diagnosis of ATTRwt-CM did not meet the current Japanese guidelines.^12^ Seven additional patients were excluded from the study because they had insufficient information for evaluation by two-dimensional speckle tracking echocardiography. Thus, data for the remaining 143 patients diagnosed with ATTRwt-CM between January 2010 and December 2021 were retrospectively analysed. We divided these patients into two groups, a cardiac death group and a non-event group, and compared the groups. Baseline clinical characteristics and echocardiographic data were obtained while the patients were in a clinically stable and non-congested condition.

This study conformed to the principles outlined in the Declaration of Helsinki. The study was approved by the institutional review board and ethics committee of Kumamoto University (approval no. 1588). The requirement for informed consent was waived because of the low-risk nature of this retrospective study and the inability to obtain consent directly from all patients. Instead, we announced the study protocol extensively at Kumamoto University Hospital and on our website (http://www2.kuh.kumamoto-u.ac.jp/tyuokensabu/index.html) and gave patients an opportunity to withdraw from the study.

### Diagnosis of ATTRwt-CM

The diagnosis of amyloid deposition was made using Congo red staining and apple-green birefringence with cross-polarized light microscopy. To confirm that the amyloid deposition was TTR, we performed immunohistochemical staining using antibodies that react with TTR. We diagnosed ATTRwt when no mutation in the *TTR* gene was revealed by genetic testing (*n* = 117, 82%) or when a patient without genetic testing had no family history of amyloidosis (*n* = 26, 18%).

ATTR-CM was diagnosed by any of the following criteria: (i) presence of TTR deposition in the myocardium (*n* = 89, 62%), (ii) presence of TTR deposition in extracardiac tissue with a positive finding on ^99m^technetium-labelled pyrophosphate scintigraphy (*n* = 27, 19%), or (iii) a positive finding on ^99m^technetium-labelled pyrophosphate scintigraphy without confirmation of pathological TTR deposition and exclusion of AL amyloidosis (*n* = 27, 19%). We excluded AL amyloidosis patients because of positive staining for immunoglobulin light chains by immunohistochemical staining and/or positive for M protein.

### Conventional echocardiographic parameters

Conventional echocardiography was performed in patients in stable condition using the Vivid E95 or 7 (GE Vingmed, Horten, Norway), Aplio 500 (Canon, Tokyo, Japan), and EPIQ 7G (Philips, Bothell, WA, USA), each of which was equipped with a 2.5 MHz phased-array transducer. Chamber size, wall thickness, LV ejection fraction (LVEF), LA volume index (LAVI), and the rate between peak early velocity of LV inflow (E velocity) and peak early diastolic velocity on the septal corner of the mitral annulus (e′) (E/e′ ratio) were evaluated using standard procedures.^13,14^ Systolic pulmonary artery pressure (sPAP) is determined from the tricuspid regurgitation (TR) jet velocity using the simplified Bernoulli equation and combining this value with an estimate of RA pressure by the diameter and collapsibility of the inferior vena cava. Tricuspid annular plane systolic excursion (TAPSE) and RV fractional area change were measured in the RV-focused apical four-chamber view. Valvular diseases were defined in accordance with the 2017 American Society of Echocardiography guideline.^15^ Moderate to severe valvular diseases defined in accordance with the guideline were included in this study. The echocardiography reviewers were blinded to the patients’ clinical histories and data to minimize evaluation bias.

### Two-dimensional strain analysis

Two-dimensional strain analysis based on speckle tracking echocardiography was performed by an operator (first operator) blinded to the clinical data and different from the operator who performed the conventional echocardiography. The two-dimensional strain analysis was performed using a vendor-independent software program (2D Strain Analysis; TOMTEC Imaging Systems, Unterschleissheim, Germany). We used averaged beats in three to five consecutive cardiac cycles as representative beats to evaluate the strain analysis. To assess RV-GLS, we evaluated the average value of the longitudinal peak systolic strain from the free wall and the septal wall of the RV in the RV-focused apical four-chamber view.^16^ We previously reported good correlation between the intra- and inter-observer variabilities for RV-GLS measurement.^8^ To assess the LV strain, the regional longitudinal strain (LS) was calculated from the echocardiographic images in the four-, three-, and two-chamber apical views. The regional LS was determined in 16 segments of the LV in accordance with the American Society for Echocardiography guidelines.^13^ The LV global LS (LV-GLS) was calculated as the average LS of these 16 segments. To assess LA LS, the regional strain was determined in three segments (septal, roof, and lateral) obtained from echocardiographic images in the four-chamber apical view in accordance with our previous report.^17,18^ To evaluate the LA strain component, the zero-strain reference was defined at end-diastole. In this study, we used LA longitudinal strain during the reservoir phase (LASr) as an indicator for the LA function because we previously revealed the prognostic utility of LA reservoir function in amyloid cardiomyopathy.^17^ Strains are described as absolute values.

### Pulmonary-RV uncoupling, LA stiffness, and VMT score

Pulmonary-RV uncoupling was estimated as the TAPSE/sPAP or RV-GLS/sPAP ratio on the basis of several previous reports.^8,9,19,20^ LA stiffness was estimated by the ratio between E/e′ and LASr.^10^ The VMT score was calculated using apical four-chamber views in early diastole and corresponding subcostal views. The time sequence of opening of the MV and TV was visually assessed in the apical four-chamber view and scored as follows: 0 = TV opening first, 1 = simultaneous valve openings, and 2 = MV opening first. When the inferior vena cava diameter was >21 mm and collapsed to <20% during normal respiration, 1 point was added, and the VMT score was calculated as 4 grades from 0 to 3.^11^

### Follow-up and prognosis

Mortality was identified by a search of the medical records and confirmed by a questionnaire and direct contact via a telephone interview with the patient or, if deceased, a family member. All deaths were reviewed and divided into cardiac or non-cardiac death. These data were confirmed in August 2023. Cardiac death was defined as death from exacerbation of heart failure or a cardiac event, or sudden death. Non-cardiac death was defined as death attributable to a non-cardiac cause.

### Statistical analysis

Continuous variables were presented as mean ± standard deviation. Categorical values were presented as number (percentage). The clinical characteristics were compared between the cardiac death group and non-event group using Student’s *t*-test, Mann–Whitney *U* test, or *χ*² test. Univariate and multivariable Cox proportional hazards analyses were performed to identify the independent parameters related to cardiac death. High-sensitivity cardiac troponin T (hs-cTnT) and B-type natriuretic peptide (BNP) concentrations were converted to log-transformed TnT and log-transformed BNP in the Cox proportional hazards analyses. Variables with possible clinical importance with a *P*-value of <0.01 in the univariate Cox hazards analysis model were incorporated into the multivariable Cox hazards analysis. Receiver operating characteristic (ROC) curves were constructed, and the areas under the curve (AUC) were calculated to assess the ability of pulmonary-RV uncoupling to predict cardiac death and to determine the associated cut-off values for predicting cardiovascular death. Differences in cardiac death predictive ability between RV-GLS/sPAP and other variables were assessed by calculating DeLong’s *P*-value, category-free net reclassification improvement (NRI), and integrated discrimination improvement (IDI) in the logistic regression models. Kaplan–Meier analysis was used to determine the cumulative incidence of cardiac death, and the log-rank test was used to compare the incidence of cardiac death between the high and low RV-GLS/sPAP groups. Statistical analyses were performed using SPSS for Windows software, version 24.0 (IBM Corp., Armonk, NY, USA), and R, version 4.0.5 (package ‘PredictABEL’; www.r-project.org). Statistical significance was defined as *P* < 0.05.

## Results

### Clinical characteristics of patients with ATTRwt-CM in the cardiac death and non-event groups

During a median follow-up of 1209 days (25th–75th percentile, 819–1527 days), 39 cardiac deaths occurred (heart failure, *n* = 38; out-of-hospital sudden death, *n* = 1). *Table 1* shows the baseline clinical characteristics, echocardiographic findings, and treatments for all patients. Patients in the cardiac death group were significantly older, comprised fewer women, and had higher rates of hospitalization for heart failure compared with the non-event group, respectively. The laboratory findings revealed that hs-cTnT and BNP concentrations were significantly higher, and estimated glomerular filtration rate (eGFR) was significantly lower, in the cardiac death group vs. the non-event group, respectively.

**Table 1:** Baseline clinical characteristics, echocardiographic findings, and treatment of ATTRwt-CM patients in this study

	Total patients (*n* = 143)	Cardiac death group (*n* = 39)	Non-event group (*n* = 104)	*P*-value
Baseline characteristics				
Age at diagnosis, years	77.2 ± 6.4	79.3 ± 6.7	76.4 ± 6.2	<0.05
Female sex, *n* (%)	16 (11)	1 (3)	15 (14)	<0.05
Body mass index, kg/m^2^	23.0 ± 4.3	23.3 ± 6.7	22.9 ± 2.9	0.57
Past medical history				
Hypertension, *n* (%)	76 (53)	22 (56)	54 (52)	0.63
Diabetes mellitus, *n* (%)	34 (24)	8 (21)	26 (25)	0.58
Dyslipidaemia, *n* (%)	47 (33)	13 (33)	34 (33)	0.94
Previous MI, *n* (%)	3 (2)	2 (5)	1 (1)	0.12
Atrial fibrillation, *n* (%)	67 (47)	20 (51)	47 (45)	0.52
Hospitalization for heart failure, *n* (%)	50 (35)	23 (59)	27 (26)	<0.01
H/CL ratio for ^99m^PYP scintigraphy	1.89 ± 0.34 (*n* = 130)	1.83 ± 0.17 (*n* = 35)	1.91 ± 0.38 (*n* = 95)	0.29
Laboratory findings				
Hs-cTnT, ng/mL	0.052 (0.036–0.080)	0.070 (0.048–0.098)	0.048 (0.031–0.072)	<0.01
B-type natriuretic peptide, pg/mL	250.7 (145.6–430.4)	330.5 (239.1–458.7)	213.8 (133.4–403.6)	<0.01
eGFR, mL/min/1.73m^2^	53.4 ± 14.4	48.3 ± 15.2	55.4 ± 13.6	<0.01
Conventional echocardiographic findings				
LAVI, mL/m^2^	58.7 ± 22.1	68.8 ± 29.5	55.0 ± 17.4	<0.01
IVSTd, mm	15.4 ± 2.5	15.8 ± 2.9	15.3 ± 2.3	0.23
LVPWTd, mm	15.7 ± 2.8	15.9 ± 2.9	15.6 ± 2.9	0.54
LVEF, %	52.6 ± 10.4	49.3 ± 10.8	53.8 ± 10.1	<0.05
E/eʹ ratio	21.3 ± 7.8	21.9 ± 8.0	21.1 ± 7.8	0.60
SPAP, mmHg	33.9 ± 11.3	39.8 ± 11.1	31.8 ± 10.6	<0.01
RVFAC, %	26.7 ± 8.4	24.7 ± 9.9	27.4 ± 7.6	0.08
TAPSE, mm	14.9 ± 5.0	14.6 ± 4.6	15.0 ± 5.2	0.67
AS (moderate, severe), *n* (%)	14 (10)	1 (3)	13 (13)	0.08
AR (moderate, severe), *n* (%)	9 (6)	3 (8)	6 (6)	0.67
MR (moderate, severe), *n* (%)	24 (17)	14 (36)	10 (10)	<0.01
TR (moderate, severe), *n* (%)	27 (19)	15 (38)	12 (12)	<0.01
Two-dimensional strain echocardiographic findings				
LV-GLS, %	9.9 ± 3.5	9.9 ± 3.0	9.8 ± 3.7	0.94
RV-GLS, %	12.1 ± 3.6	11.0 ± 3.7	12.5 ± 3.6	<0.05
LASr, %	7.49 ± 4.39	6.57 ± 3.31	7.84 ± 4.70	0.13
New echocardiographic factors				
TAPSE/sPAP	0.44 (0.30–0.61)	0.40 (0.25–0.47)	0.48 (0.32–0.65)	<0.01
RV-GLS/sPAP	0.35 (0.26–0.53)	0.29 (0.18–0.35)	0.40 (0.29–0.57)	<0.01
E/eʹ/LALS	4.64 ± 5.84	4.5 ± 3.5	4.7 ± 6.5	0.90
VMT score	1.20 ± 0.62	1.31 ± 0.61	1.16 ± 0.63	0.22
VMT score ≥ 2	41 (29)	13 (33)	28 (27)	0.45
Treatments				
ACEI or ARB, *n* (%)	58 (41)	19 (49)	39 (38)	0.22
MRA, *n* (%)	40 (28)	14 (36)	26 (25)	0.20
β-Blocker, *n* (%)	39 (27)	13 (33)	26 (25)	0.32
Diuretics, *n* (%)	94 (66)	34 (87)	60 (58)	<0.01
Tafamidis, *n* (%)	73 (51)	9 (23)	64 (62)	<0.01

The *P*-values were obtained by Student’s *t*-test, Mann–Whitney *U* test, or *χ*² test.

ATTRwt-CM, wild-type transthyretin amyloid cardiomyopathy; MI, myocardial infarction; H/CL ratio, heart to contralateral ratio; ^99m^PYP scintigraphy, 99 m technetium-pyrophosphate scintigraphy; hs-cTnT, high-sensitivity cardiac troponin T; eGFR, estimated glomerular filtration rate; LAVI, left atrial volume index; IVSTd, interventricular septal thickness in diastole; LVPWTd, left ventricular posterior wall thickness in diastole; LVEF, left ventricular ejection fraction; SPAP, systolic pulmonary artery pressure; RVFAC, right ventricular fractional area change; TAPSE, tricuspid annular plane systolic excursion; AS, aortic stenosis; AR, aortic regurgitation; MR, mitral regurgitation; TR, tricuspid regurgitation; LV-GLS, left ventricular-global longitudinal strain; RV-GLS, right ventricular-global longitudinal strain; LASr, left atrial longitudinal strain during the reservoir phase; VMT score, visually assessed time difference between mitral valve and tricuspid valve opening score; ACEI, angiotensin-converting enzyme inhibitor; ARB, angiotensin receptor blocker; MRA, mineralocorticoid receptor antagonist.

In the conventional echocardiographic findings, LAVI, sPAP, and the rates of mitral regurgitation (MR) and TR were significantly higher, and LVEF was significantly lower, in the cardiac death group vs. the non-event group, respectively. For the two-dimensional strain echocardiographic findings, only RV-GLS was significantly lower in patients in the cardiac death group vs. the non-event group. Among the new echocardiographic factors, TAPSE/sPAP and RV-GLS/sPAP were significantly lower in the cardiac death group vs. the non-event group, respectively. Among the treatment regimens, the rate of diuretics use was significantly higher, and the rate of tafamidis use was significantly lower, in the cardiac death group vs. the non-event group, respectively.

### Cox proportional hazards regression analysis for cardiac death in patients with ATTRwt-CM

As shown in *Table 2*, univariate Cox proportional hazards regression analysis showed that 17 variables were significantly associated with cardiac death: age, previous myocardial infarction, hospitalization for heart failure, hs-cTnT, BNP, eGFR, LAVI, LVEF, sPAP, MR, TR, RV-GLS, TAPSE/sPAP, RV-GLS/sPAP, VMT score, tafamidis use, and diuretics use. Considering the internal correlation and the number of patients in our study, we created six models to perform the multivariate Cox proportional hazards regression analysis (*Tables 3* and *4*). RV-GLS/sPAP was significantly and independently associated with cardiac death after adjusting for TAPSE/sPAP (Model 1), sPAP (Model 2), conventional prognostic factors (Model 3), prognostic laboratory factors (Model 4), prognostic echocardiographic factors (Model 5), and treatment regimens (Model 6).

**Table 2:** Univariate Cox proportional hazards model for cardiac death

	Univariate analysis	
	HR (95% CI)	*P*-value
Age per 1 year increment	1.12 (1.05–1.19)	<0.01
Female sex	0.38 (0.05–2.77)	0.34
Body mass index per 1 kg/m^2^	1.00 (0.92–1.10)	0.95
Hypertension/yes	1.15 (0.61–2.17)	0.67
Diabetes mellitus/yes	1.00 (0.46–2.20)	0.99
Dyslipidaemia/yes	1.24 (0.63–2.43)	0.53
Previous MI/yes	6.37 (1.49–27.33)	<0.05
Atrial fibrillation/yes	1.46 (0.78–2.76)	0.24
Hospitalization for heart failure/yes	4.52 (2.30–8.86)	<0.01
Log-transformed TnT/1	3.30 (1.87–5.83)	<0.01
Log-transformed BNP/1	2.78 (1.60–4.81)	<0.01
eGFR/1 mL/min/1.73m^2^	0.95 (0.93–0.98)	<0.01
LAVI/1 mL/m^2^	1.02 (1.01–1.03)	<0.01
IVSTd/1mm	1.00 (0.89–1.13)	0.99
LVPWTd/1mm	0.99 (0.89–1.11)	0.87
LVEF/1%	0.97 (0.94–1.00)	<0.05
E/eʹ ratio/1	1.03 (0.99–1.07)	0.19
SPAP/1mmHg	1.07 (1.04–1.10)	<0.01
RVFAC/1%	0.97 (0.93–1.00)	0.05
TAPSE/1mm	0.97 (0.90–1.04)	0.35
AS (moderate, severe)/yes	0.59 (0.08–4.38)	0.61
AR (moderate, severe)/yes	1.79 (0.54–5.88)	0.34
MR (moderate, severe)/yes	2.68 (1.39–5.17)	<0.01
TR (moderate, severe)/yes	3.63 (1.88–7.03)	<0.01
LV-GLS/1%	0.95 (0.86–1.05)	0.31
RV-GLS/1%	0.89 (0.81–0.98)	<0.05
LASr/1%	0.93 (0.85–1.01)	0.07
TAPSE/sPAP/1 mm/mmHg	0.07 (0.01–0.42)	<0.01
RV-GLS/sPAP/1 mm/mmHg	0.01 (0.00–0.07)	<0.01
E/eʹ/LASr/1	1.01 (0.96–1.06)	0.78
VMT score/1	1.72 (1.04–2.86)	<0.05
Tafamidis/yes	0.10 (0.04–0.24)	<0.01
ACEI or ARB/yes	1.03 (0.54–1.96)	0.93
MRA/yes	1.42 (0.74–2.74)	0.30
Beta blocker/yes	0.97 (0.49–1.92)	0.94
Diuretic agent/yes	4.23 (1.65–10.84)	<0.01

*P*-value was obtained by the univariate Cox hazards analysis model.

MI, myocardial infarction; TnT, troponin T; BNP, B-type natriuretic peptide; eGFR, estimated glomerular filtration rate; LAVI, left atrial volume index; IVSTd, interventricular septal thickness in diastole; LVPWTd, left ventricular posterior wall thickness in diastole; LVEF, left ventricular ejection fraction; SPAP, systolic pulmonary artery pressure; RVFAC, right ventricular fractional area change; TAPSE, tricuspid annular plane systolic excursion; AS, aortic stenosis; AR, aortic regurgitation; MR, mitral regurgitation; TR, tricuspid regurgitation; LV-GLS, left ventricular-global longitudinal strain; RV-GLS, right ventricular-global longitudinal strain; LASr, left atrial longitudinal strain during the reservoir phase; VMT score, visually assessed time difference between mitral valve and tricuspid valve opening score; ACEI, angiotensin-converting enzyme inhibitor; ARB, angiotensin receptor blocker; MRA, mineralocorticoid receptor antagonist.

**Table 3:** Multivariable Cox proportional hazards model for cardiac death

	Model 1		Model 2		Model 3	
	HR (95% CI)	*P*-value	HR (95% CI)	*P*-value	HR (95% CI)	*P*-value
RV-GLS/sPAP per 1%/mmHg	0.00 (0.00–0.11)	<0.01	0.03 (0.00–0.64)	<0.05	0.01 (0.00–0.13)	<0.01
TAPSE/sPAP per 1 mm/mmHg	2.07 (0.15–29.27)	0.59				
SPAP per 1mmHg			1.03 (0.99–1.08)	0.17		
Age per 1 year					1.16 (1.08–1.23)	<0.01
Hospitalization for heart failure/yes					2.76 (1.39–5.89)	<0.01


*P*-value was obtained by the multivariate Cox hazards analysis.

RV-GLS, right ventricular-global longitudinal strain; SPAP, systolic pulmonary artery pressure; TAPSE, tricuspid annular plane systolic excursion.

**Table 4:** Multivariable Cox proportional hazards model for cardiovascular death

	Model 4		Model 5		Model 6	
	HR (95% CI)	*P*-value	HR (95% CI)	*P*-value	HR (95% CI)	*P*-value
RV-GLS/TRPG per 1%/mmHg	0.02 (0.00–0.25)	<0.01	0.02 (0.00–0.34)	<0.01	0.02 (0.00–0.23)	<0.01
Log-transformed TnT/1	1.48 (0.70–3.11)	0.30				
Log-transformed BNP/1	1.41 (0.71–2.81)	0.33				
eGFR/1 mL/min/1.73m^2^	0.97 (0.94–1.00)	0.09				
LAVI/1 mL/m^2^			1.01 (0.99–1.02)	0.29		
MR (moderate, severe)/yes			1.50 (0.71–3.16)	0.29		
TR (moderate, severe)/yes			1.36 (0.59–3.12)	0.47		
Tafamidis/yes					0.11 (0.04–0.27)	<0.01
Diuretic agent/yes					1.52 (0.51–4.58)	0.46

*P*-value was obtained by the multivariate Cox hazards analysis.

RV-GLS, right ventricular-global longitudinal strain; TnT, troponin T; BNP, B-type natriuretic peptide; eGFR, estimated glomerular filtration rate; LAVI, left atrial volume index; MR, mitral regurgitation; TR, tricuspid regurgitation.

### ROC analysis for cardiovascular death

**Figure 1:**
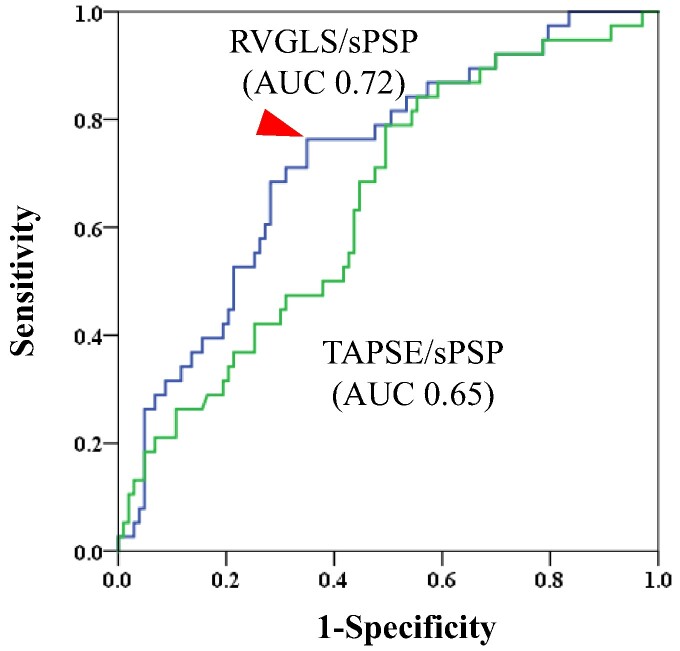
Receiver operator characteristic curve analysis of RV-GLS/sPAP (blue line) and TAPSE/sPAP (green line) to predict cardiac death. Red arrow indicates cut-off point. RV-GLS, right ventricular global longitudinal strain; sPAP, systolic pulmonary artery pressure; TAPSE, tricuspid annular plane systolic excursion; AUC, area under the curve.

*Figure 1* shows the results of the ROC analysis of RV-GLS/sPAP and TAPSE/sPAP for cardiac death. The AUC of RV-GLS/sPAP for cardiac death was 0.72. In contrast, the AUCs of TAPSE/sPAP were 0.65. We found that the best cut off value for RV-GLS/sPAP was 0.34 (sensitivity: 76%; specificity: 65%; red arrow; *Figure 1*).

### DeLong’s *P*-value, NRI, and IDI in the logistic regression model

The addition of RV-GLS/sPAP to TAPSE/sPAP, E/e′/LASr, or VMT score significantly improved reclassification of the risk of cardiac death in patients with ATTRwt-CM on the basis of the DeLong’s *P*-value and NRI and IDI values (*Table 5*).

**Table 5:** DeLong’s *P*-value, NRI, and IDI in logistic model

RV-GLS/sPAP	DeLong’s *P*-value	NRI (95% CI)	*P*-value	IDI (95% CI)	*P*-value
TAPSE/sPAP	<0.05	0.14 (0.04–0.23)	<0.01	0.06 (0.03–0.09)	<0.01
E/eʹ/LASr	<0.01	0.17 (0.08–0.26)	<0.01	0.10 (0.05–0.15)	<0.01
VMT score	<0.01	0.17 (0.08–0.26)	<0.01	0.08 (0.04–0.13)	<0.01

NRI, net reclassification improvement; IDI, integrated discrimination improvement; RV-GLS, right ventricular-global longitudinal strain; sPAP, systolic pulmonary artery pressure; TAPSE, tricuspid annular plane systolic excursion; LASr, left atrial longitudinal strain during the reservoir phase; VMT score, visually assessed time difference between mitral valve and tricuspid valve opening score.

### Follow-up of patients with ATTRwt-CM

**Figure 2:**
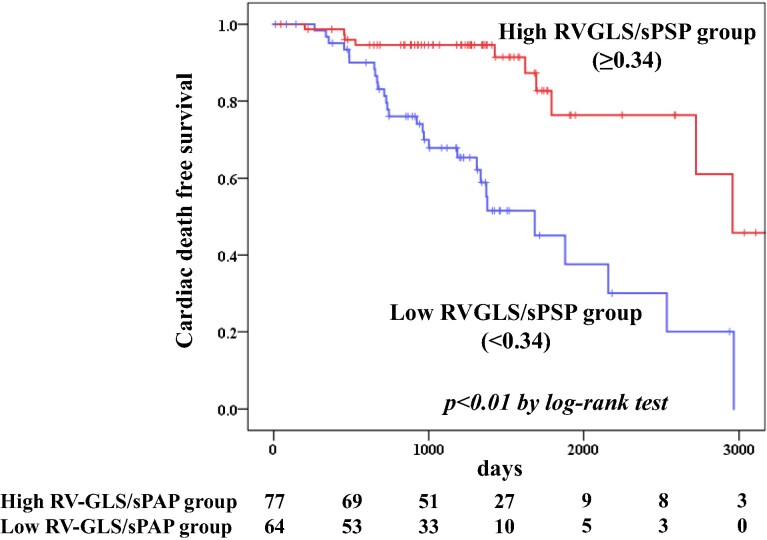
Kaplan–Meier curves of cardiac death in patients with ATTRwt-CM with high or low RV-GLS/sPAP. ATTRwt-CM, wild-type transthyretin amyloid cardiomyopathy; RV-GLS, right ventricular global longitudinal strain, sPAP, systolic pulmonary artery pressure; *P*-value was obtained by log-rank test.

We divided the patients with ATTRwt-CM into a low RV-GLS/sPAP group (<0.34, *n* = 64) and high RV-GLS/sPAP group (≥0.34, *n* = 77) using the best cut off value of RV-GLS/sPAP (0.34) as estimated by the ROC curve analysis. Kaplan–Meier analysis demonstrated a significantly higher probability of cardiac death (*P* < 0.01 by the log-rank test) (*Figure 2*) in patients in the low RV-GLS/sPAP group compared with the high RV-GLS/sPAP group.

## Discussion

In this study, both RV-GLS and sPAP were associated with cardiac death in patients with ATTRwt-CM. However, pulmonary-RV uncoupling was superior to both RV-GLS and sPAP alone. In left-sided heart failure, LV dysfunction secondarily aggravates RV dysfunction because of ventricular interdependence, neurohormonal interactions, myocardial ischaemia of the RV, and pulmonary hypertension.^21^ Of these, pulmonary hypertension is the primary mechanism of RV failure.^22^ Several studies have shown that pulmonary hypertension and RV failure are independently associated with clinical worsening.^23,24^ Additionally, amyloid deposition can occur in both the LV and RV,^25^ which leads to RV dysfunction. In amyloid cardiomyopathy, amyloid deposition is another important mechanism of RV dysfunction. Amyloid deposition weakens the response of the RV to pulmonary hypertension, which might explain why pulmonary-RV uncoupling significantly predicted cardiac death in patients with ATTRwt-CM, in our study.

Guazzi et al.^9^ proposed an index for pulmonary-RV uncoupling assessed by the ratio of longitudinal RV fibre shortening (TAPSE) to developed pressure (sPAP). Palmiero et al.^26^ showed that TAPSE/sPAP was an independent predictor of cardiovascular death and had potential to improve risk stratification and guide management strategies in patients with amyloid cardiomyopathy. However, our present study revealed that RV-GLS/sPAP was more useful than TAPSE/sPAP to predict the prognosis of ATTRwt-CM. A previous report showed a correlation between TAPSE, an easily obtainable parameter of RV longitudinal function, with parameters used to estimate RV global systolic function.^13^ However, TAPSE is affected by LV apical motion and has a tendency to over- or under-estimate RV function relative to the transducer position because this index is an angle-dependent one-dimensional measurement.^20^ These points might explain why RV-GLS/sPAP was a more useful prognostic marker than TAPSE/sPAP in our study.

LA stiffness has recently gained much attention. LA stiffness can be assessed by the ratio of change in LA pressure to volume during passive filling of the LA. When LA strain is used in conjunction with invasively measured pulmonary artery wedge pressure or E/e′, LA stiffness can be derived. Increased LA stiffness is associated with an increased risk of all-cause mortality and hospitalization caused by heart failure in patients with heart failure-preserved EF.^27^ However, in our study, LA stiffness was not an important prognostic factor in patients with ATTRwt-CM. The cut-off point of the LA stiffness index (E/e′/LASr) in a previous study was 0.26.^27^ In contrast, the average E/e′/LASr was >4.0 in our study. There was a dramatic difference in LA stiffness between the results in the previous study and our study. LA dysfunction is usually correlated with greater impairment of LV diastolic function because higher LV filling pressure leads to deterioration of LA function as a result of haemodynamic overload and stretching of the LA wall.^28^ In contrast, in our study, LA function in amyloid cardiomyopathy was affected by both LV diastolic function and direct amyloid deposition. LA function was severely impaired in almost all patients with amyloid cardiomyopathy in both the cardiac death group and the non-event group. This might explain why LA stiffness was not a significant predictive factor in patients with ATTRwt-CM in our study.

When LV filling pressure increases, the MV opens early and precedes TV opening in early diastole. Murayama et al.^11^ named the visually assessed time sequence of atrioventricular valve opening as the VMT score and reported the score as a new marker of elevated LV filling pressure with important prognostic utility in patients with heart failure. However, the VMT score did not have significant usefulness to predict cardiac death in our study. We previously reported that various echocardiographic markers related to LA function, RV function, and RA function were useful to predict prognosis in patients with amyloid cardiomyopathy.^8,17,29^ However, in these previous studies, echocardiographic markers related to LV function were not statistically significantly useful to predict prognosis, indicating that LV function was relatively unimportant to predict the prognosis in patients with ATTRwt-CM. These findings might also explain why VMT score, one of the indices of LV function, was not an important prognostic marker of ATTRwt-CM in our study.

## Study limitations

This study has several limitations. First, this study involved a small number of patients and was performed at a single centre. Second, several patients were diagnosed with ATTRwt-CM before the RV-focused apical four-chamber view was recommended. Therefore, we had no choice but to use the apical four-chamber view to evaluate RV-GLS in these patients. Additionally, the e′ lateral line was not measured in several patients because the patients were diagnosed with ATTRwt-CM before the e′ lateral line was recommended. Therefore, we used only the e′ septal line to evaluate LV diastolic function and LA stiffness in the study. Third, we obtained echocardiographic images using several brands of ultrasound machine. Furthermore, the two-dimensional speckle tracking echocardiography analysis was performed with TOMTEC Image-Arena™ (vendor-independent) software. Although there are significant correlations in the LS values analysed using vendor-independent software for paired images obtained from ultrasound machines from different manufacturers,^30^ inter-manufacturer variability exists and might have affected our study results.

Despite these limitations, our study is unique and the first to demonstrate the importance of pulmonary-RV uncoupling evaluated by RV-GLS/sPAP in patients with ATTRwt-CM. We believe that our results have significant clinical value.

## Conclusion

Pulmonary-RV uncoupling has prognostic value in patients with ATTRwt-CM and provides greater prognostic power compared with conventional prognostic factors.

## Data Availability

The data underlying the research results described in this paper are not publicly available due to the privacy of the research participants’ data.

## Lead author biography

Hiroki Usuku is a cardiologist at Kumamoto University Hospital, Kumamoto, Japan. His research focuses on echocardiography. A main focus of his research is to evaluate right ventricular and atrial functions by two-dimensional strain echocardiography in patients with heart failure, pulmonary hypertension, and amyloid cardiomyopathy.

## References

[1] Falk RH, Dubrey SW. Amyloid heart disease. Prog Cardiovasc Dis 2010;52:347–61. 20109604 10.1016/j.pcad.2009.11.007

[2] Marume K, Takashio S, Nishi M, Hirakawa K, Yamamoto M, Hanatani S et al Combination of commonly examined parameters is a useful predictor of positive (99 m)Tc-labeled pyrophosphate scintigraphy findings in elderly patients with suspected transthyretin cardiac amyloidosis. Circ J 2019;83:1698–708. 31189791 10.1253/circj.CJ-19-0255

[3] Castano A, Drachman BM, Judge D, Maurer MS. Natural history and therapy of TTR-cardiac amyloidosis: emerging disease-modifying therapies from organ transplantation to stabilizer and silencer drugs. Heart Fail Rev 2015;20:163–78. 25408161 10.1007/s10741-014-9462-7 PMC4361302

[4] Grogan M, Scott CG, Kyle RA, Zeldenrust SR, Gertz MA, Lin G et al Natural history of wild-type transthyretin cardiac amyloidosis and risk stratification using a novel staging system. J Am Coll Cardiol 2016;68:1014–20. 27585505 10.1016/j.jacc.2016.06.033

[5] Gillmore JD, Damy T, Fontana M, Hutchinson M, Lachmann HJ, Martinez-Naharro A et al A new staging system for cardiac transthyretin amyloidosis. Eur Heart J 2018;39:2799–806. 29048471 10.1093/eurheartj/ehx589

[6] Amundsen BH, Helle-Valle T, Edvardsen T, Torp H, Crosby J, Lyseggen E et al Noninvasive myocardial strain measurement by speckle tracking echocardiography: validation against sonomicrometry and tagged magnetic resonance imaging. J Am Coll Cardiol 2006;47:789–93. 16487846 10.1016/j.jacc.2005.10.040

[7] Monte IP, Faro DC, Trimarchi G, de Gaetano F, Campisi M, Losi V et al Left atrial strain imaging by speckle tracking echocardiography: the supportive diagnostic value in cardiac amyloidosis and hypertrophic cardiomyopathy. J Cardiovasc Dev Dis 2023;10:261. 37367426 10.3390/jcdd10060261 PMC10299603

[8] Usuku H, Takashio S, Yamamoto E, Yamada T, Egashira K, Morioka M et al Prognostic value of right ventricular global longitudinal strain in transthyretin amyloid cardiomyopathy. J Cardiol 2022;80:56–63. 35282945 10.1016/j.jjcc.2022.02.010

[9] Guazzi M, Bandera F, Pelissero G, Castelvecchio S, Menicanti L, Ghio S et al Tricuspid annular plane systolic excursion and pulmonary arterial systolic pressure relationship in heart failure: an index of right ventricular contractile function and prognosis. Am J Physiol Heart Circ Physiol 2013;305:H1373–1381. 23997100 10.1152/ajpheart.00157.2013

[10] Kurt M, Wang J, Torre-Amione G, Nagueh SF. Left atrial function in diastolic heart failure. Circ Cardiovasc Imaging 2009;2:10–5. 19808559 10.1161/CIRCIMAGING.108.813071

[11] Murayama M, Iwano H, Nishino H, Tsujinaga S, Nakabachi M, Yokoyama S et al Simple two-dimensional echocardiographic scoring system for the estimation of left ventricular filling pressure. J Am Soc Echocardiogr 2021;34:723–34. 33675942 10.1016/j.echo.2021.02.013

[12] Kitaoka H, Izumi C, Izumiya Y, Inomata T, Ueda M, Kubo T et al JCS 2020 guideline on diagnosis and treatment of cardiac amyloidosis. Circ J 2020;84:1610–71. 32830187 10.1253/circj.CJ-20-0110

[13] Lang RM, Badano LP, Mor-Avi V, Afilalo J, Armstrong A, Ernande L et al Recommendations for cardiac chamber quantification by echocardiography in adults: an update from the American Society of Echocardiography and the European Association of Cardiovascular Imaging. J Am Soc Echocardiogr 2015;28:1–39.e14. 25559473 10.1016/j.echo.2014.10.003

[14] Nagueh SF, Smiseth OA, Appleton CP, Byrd BF III, Dokainish H, Edvardsen T et al Recommendations for the evaluation of left ventricular diastolic function by echocardiography: an update from the American Society of Echocardiography and the European Association of Cardiovascular Imaging. J Am Soc Echocardiogr 2016;29:277–314. 27037982 10.1016/j.echo.2016.01.011

[15] Zoghbi WA, Adams D, Bonow RO, Enriquez-Sarano M, Foster E, Grayburn PA et al Recommendations for noninvasive evaluation of native valvular regurgitation: a report from the American Society of Echocardiography developed in collaboration with the Society for Cardiovascular Magnetic Resonance. J Am Soc Echocardiogr 2017;30:303–71. 28314623 10.1016/j.echo.2017.01.007

[16] Badano LP, Kolias TJ, Muraru D, Abraham TP, Aurigemma G, Edvardsen T et al Standardization of left atrial, right ventricular, and right atrial deformation imaging using two-dimensional speckle tracking echocardiography: a consensus document of the EACVI/ASE/Industry Task Force to standardize deformation imaging. Eur Heart J Cardiovasc Imaging 2018;19:591–600. 29596561 10.1093/ehjci/jey042

[17] Oike F, Usuku H, Yamamoto E, Yamada T, Egashira K, Morioka M et al Prognostic value of left atrial strain in patients with wild-type transthyretin amyloid cardiomyopathy. ESC Heart Fail 2021;8:5316–26. 34582129 10.1002/ehf2.13621 PMC8712780

[18] Oike F, Usuku H, Yamamoto E, Marume K, Takashio S, Ishii M et al Utility of left atrial and ventricular strain for diagnosis of transthyretin amyloid cardiomyopathy in aortic stenosis. ESC Heart Fail 2022;9:1976–86. 35338611 10.1002/ehf2.13909 PMC9065867

[19] Magnino C, Omedè P, Avenatti E, Presutti D, Iannaccone A, Chiarlo M et al Inaccuracy of right atrial pressure estimates through inferior vena cava indices. Am J Cardiol 2017;120:1667–73. 28912040 10.1016/j.amjcard.2017.07.069

[20] Giusca S, Dambrauskaite V, Scheurwegs C, D’Hooge J, Claus P, Herbots L et al Deformation imaging describes right ventricular function better than longitudinal displacement of the tricuspid ring. Heart 2010;96:281–8. 19720609 10.1136/hrt.2009.171728

[21] Haddad F, Doyle R, Murphy DJ, Hunt SA. Right ventricular function in cardiovascular disease, part II: pathophysiology, clinical importance, and management of right ventricular failure. Circulation 2008;117:1717–31. 18378625 10.1161/CIRCULATIONAHA.107.653584

[22] Guazzi M, Galiè N. Pulmonary hypertension in left heart disease. Eur Respir Rev 2012;21:338–46. 23204122 10.1183/09059180.00004612 PMC9487233

[23] Ghio S, Gavazzi A, Campana C, Inserra C, Klersy C, Sebastiani R et al Independent and additive prognostic value of right ventricular systolic function and pulmonary artery pressure in patients with chronic heart failure. J Am Coll Cardiol 2001;37:183–8. 11153735 10.1016/s0735-1097(00)01102-5

[24] Ghio S, Temporelli PL, Klersy C, Simioniuc A, Girardi B, Scelsi L et al Prognostic relevance of a non-invasive evaluation of right ventricular function and pulmonary artery pressure in patients with chronic heart failure. Eur J Heart Fail 2013;15:408–14. 23307814 10.1093/eurjhf/hfs208

[25] Falk RH . Diagnosis and management of the cardiac amyloidoses. Circulation 2005;112:2047–60. 16186440 10.1161/CIRCULATIONAHA.104.489187

[26] Palmiero G, Monda E, Verrillo F, Dongiglio F, Caiazza M, Rubino M et al Prevalence and clinical significance of right ventricular pulmonary arterial uncoupling in cardiac amyloidosis. Int J Cardiol 2023;388:131147. 37423570 10.1016/j.ijcard.2023.131147

[27] Kim D, Seo JH, Choi KH, Lee SH, Choi JO, Jeon ES et al Prognostic implications of left atrial stiffness Index in heart failure patients with preserved ejection fraction. JACC Cardiovasc Imaging 2023;16:435–45. 36752431 10.1016/j.jcmg.2022.11.002

[28] Guan Z, Zhang D, Huang R, Zhang F, Wang Q, Guo S. Association of left atrial myocardial function with left ventricular diastolic dysfunction in subjects with preserved systolic function: a strain rate imaging study. Clin Cardiol 2010;33:643–9. 20960540 10.1002/clc.20784 PMC6653575

[29] Usuku H, Yamamoto E, Sueta D, Shinriki R, Oike F, Tabata N et al A new staging system using right atrial strain in patients with immunoglobulin light-chain cardiac amyloidosis. ESC Heart Fail 2024;11:1612–24. 38400613 10.1002/ehf2.14710 PMC11098642

[30] Nagata Y, Takeuchi M, Mizukoshi K, Wu VC, Lin FC, Negishi K et al Intervendor variability of two-dimensional strain using vendor-specific and vendor-independent software. J Am Soc Echocardiogr 2015;28:630–41. 25747915 10.1016/j.echo.2015.01.021

